# P-2198. High Prevalence of HBeAg negative among People Living with HIV and Hepatitis B ART naïve in Maputo - Mozambique

**DOI:** 10.1093/ofid/ofae631.2352

**Published:** 2025-01-29

**Authors:** Lucia Mabalane Chambal, Charlotta Nilsson, Elias Manjate, Corssino Tchavana, Orvalho Augusto, Esperanca Sevene Comiche

**Affiliations:** Eduardo Mondlane University, Maputo, Maputo, Mozambique; Karolinska Institutet, Stockholm, Stockholms Lan, Sweden; Eduardo Mondlane University, Maputo, Maputo, Mozambique; Manhiça Health Research Center (CISM), Maputo, Maputo, Mozambique; University of Washington, Seatle, Washington; Eduardo Mondlane University, Maputo, Maputo, Mozambique

## Abstract

**Background:**

About 7.4% of the people living with HIV in the world are co-infected with hepatitis B virus (HBV), and more than 70% of the co-infected reside in Africa. HBeAg-negative chronic hepatitis B (CHB) is associated with persistent low viremia and progression into cirrhosis with a 3-10% yearly rate and with rare spontaneous remissions. We report the frequency of HBeAg among HIV/HBV coinfected recently diagnosed ART naïve patients.

**Methods:**

We analyzed baseline data of a prospective cohort of newly HIV-diagnosed ART naïve patients at Mavalane Health Centre located in a periurban area in Maputo City. Between April 2021 and November 2022, all patients acove 18 years of age were enrolled then screened for HBsAg. All participants had CD4+ T cells count, further serological markers of hepatitis B (anti-HBc IgM, HBeAg, anti-HBe), HIV and HBV viral loads assessed using standard procedures. Quantitative variables were expressed as mean and standard deviation, whereas qualitative ones were expressed as frequency and percentage.

**Results:**

A total of 1,106 participants were included, and HBsAg was reactive in 81 participants, yielding a co-infection rate of 7.3%. Being male (OR = 0.58 and 95%CI: 0.59 to 0.94) or sex worker (OR = 3.69, 95%CI: 1.10 to 10.58) was associated with being co-infected. Overall, 67/81 (80.2%) of the co-infected were HBeAg negative and 14/81 (19.8%) were HBeAg positive. The median APRI was 0.5 (IQR 0.3, 1.1) for the HBeAg negative and 0.7 (0.3, 1.4) for the HBeAg positive. The median HBV-DNA was 258.0 IU (IQR 10.0, 4,974.5) for the HBeAg negative and 746,287.0 IU (IQR 2,720.0, 49,899,213.0) for the HBeAg positive subjects (p < 0.001).Among the HBeAg negative 44 (65.7%) had a detectable HBV-DNA with 20 (29.9%) presenting with HBV-DNA > 2000 IU and 14 (20.9%) with a HBV-DNA > 20 000 UI. Two (3.0%) HBeAg negative participants and one (7.1%) HBeAg positive participants presented with hepatocellular carcinoma (HCC).

**Conclusion:**

These results confirm the high prevalence of HIV and HBV co-infection in Mozambique and brings new data on HBeAg negative frequency. There is a need to test for HBV all HIV infected and integrate the management and monitoring of hepatitis B and liver disease-specific tests in public ART programs to predict and reduce the occurrence of HBV complications and mortality.

**Disclosures:**

All Authors: No reported disclosures

